# Supplementary figures and images for: Integration of whole genome sequencing and transcriptomics reveals a complex picture of the reestablishment of insecticide resistance in the major malaria vector Anopheles coluzzii
Source: PLoS Genet. 2021 Dec 23;17(12):e1009970. doi: 10.1371/journal.pgen.1009970 (PMC8741062; doi:10.1371/journal.pgen.1009970)

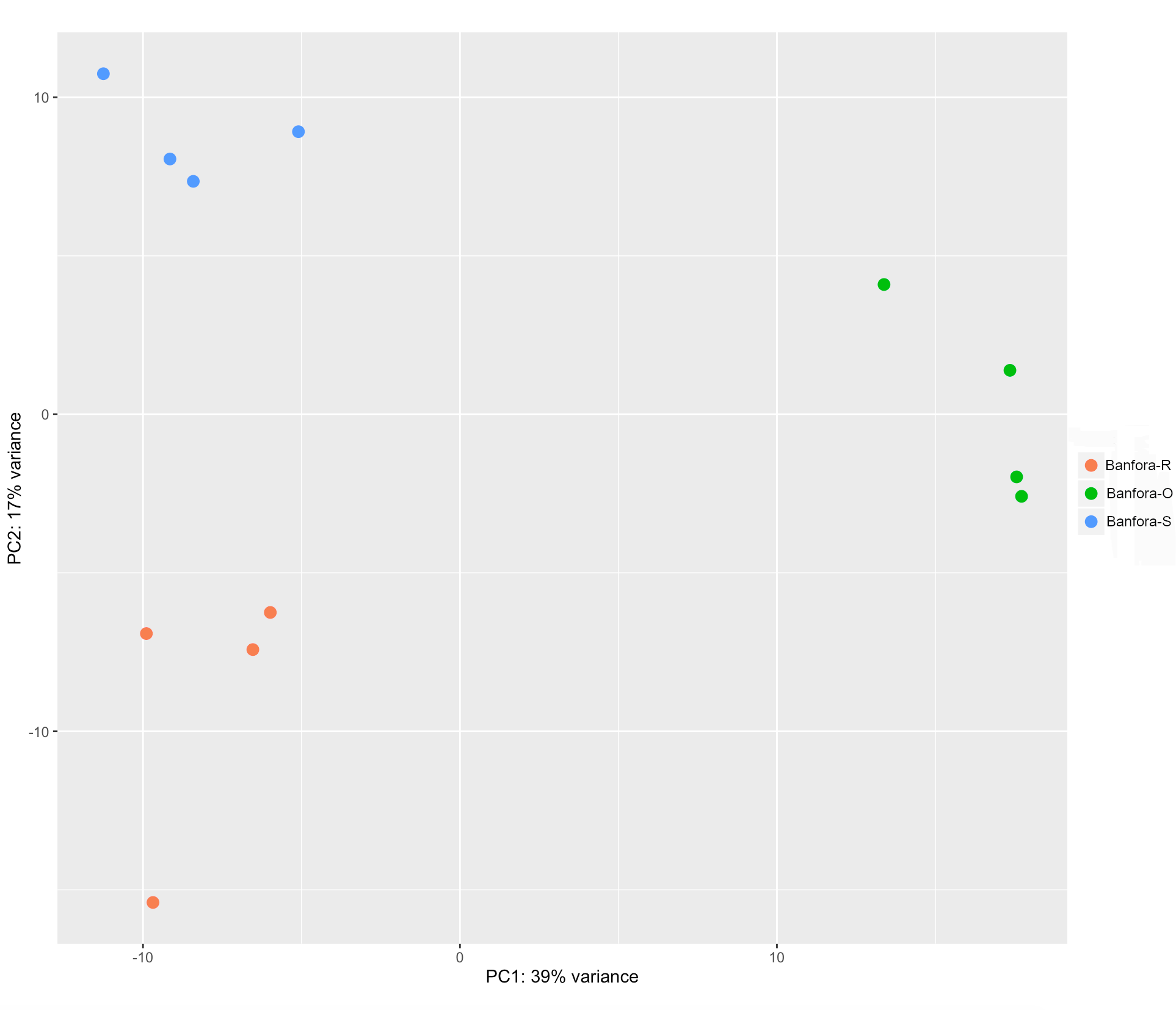

Supplement: S1 Fig — PCA performed on variance stabilising transformation on count data using DEseq2. (TIF) [file pgen.1009970.s001.tif]

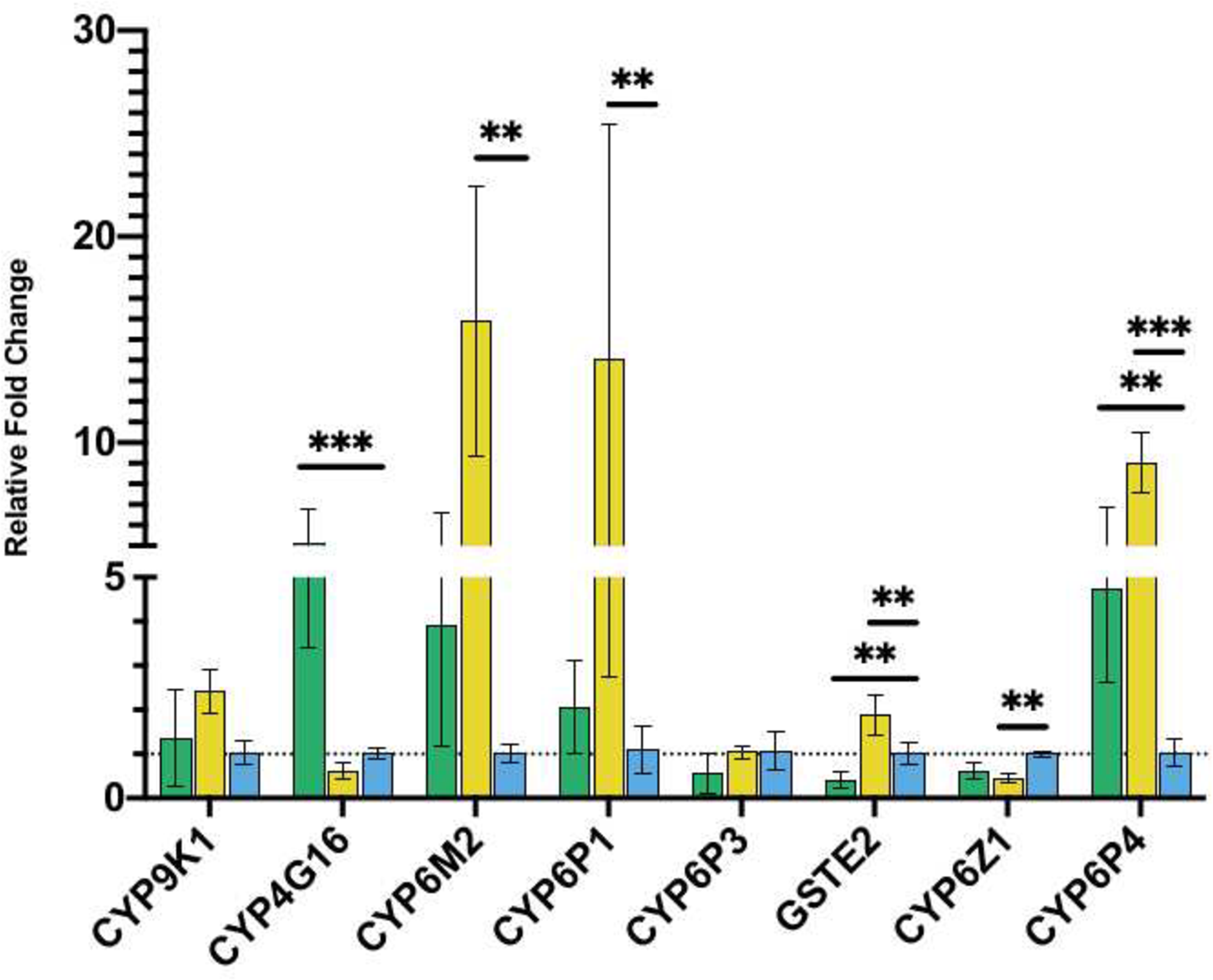

Supplement: S2 Fig — Relative mRNA expression levels between the original (green) and susceptible (blue) and re-selected (orange) and susceptible (blue) for 8 genes previously linked with resistance. Significance was calculated by an ANOVA followed by Dunnett’s multiple testing. Adjusted p values are shown with significance as follows: ** p < 0.01 and *** p < 0.001. (TIF) [file pgen.1009970.s002.tif]

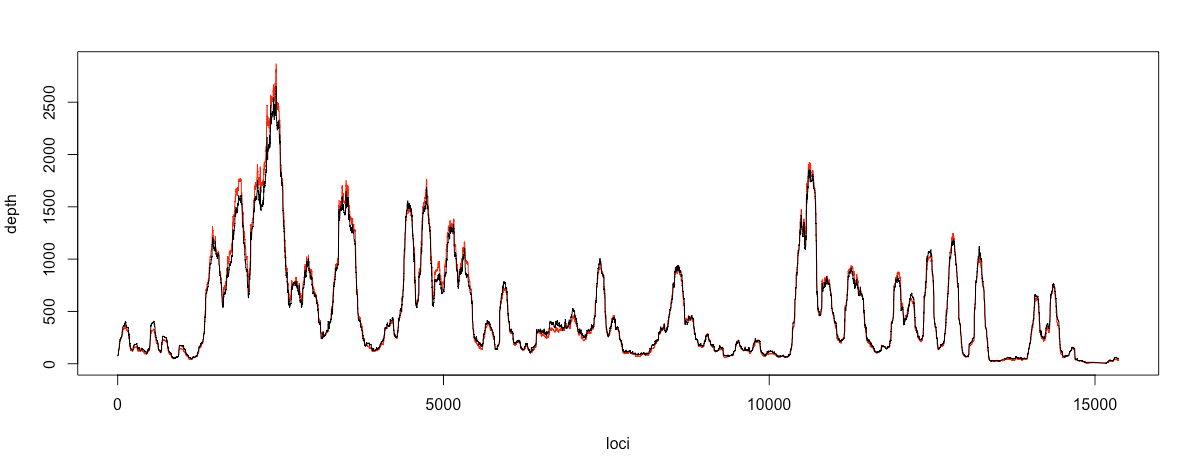

Supplement: S3 Fig — Read depth (y) along the full mitochondrial genome (x) for the re-selected (red) and susceptible (black). (TIF) [file pgen.1009970.s003.tif]

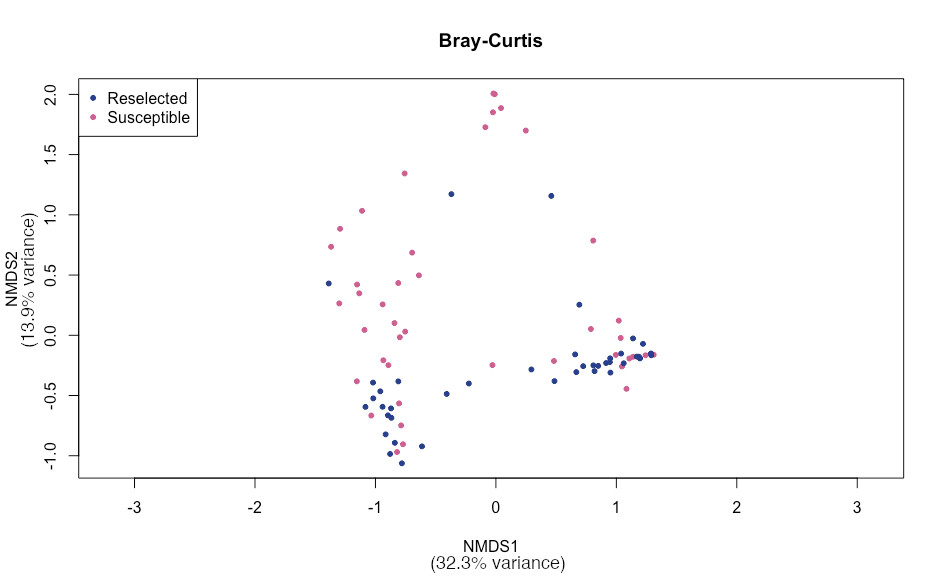

Supplement: S4 Fig — Graph showing the similarities of the different samples in terms of microbe abundance. (TIF) [file pgen.1009970.s004.tif]

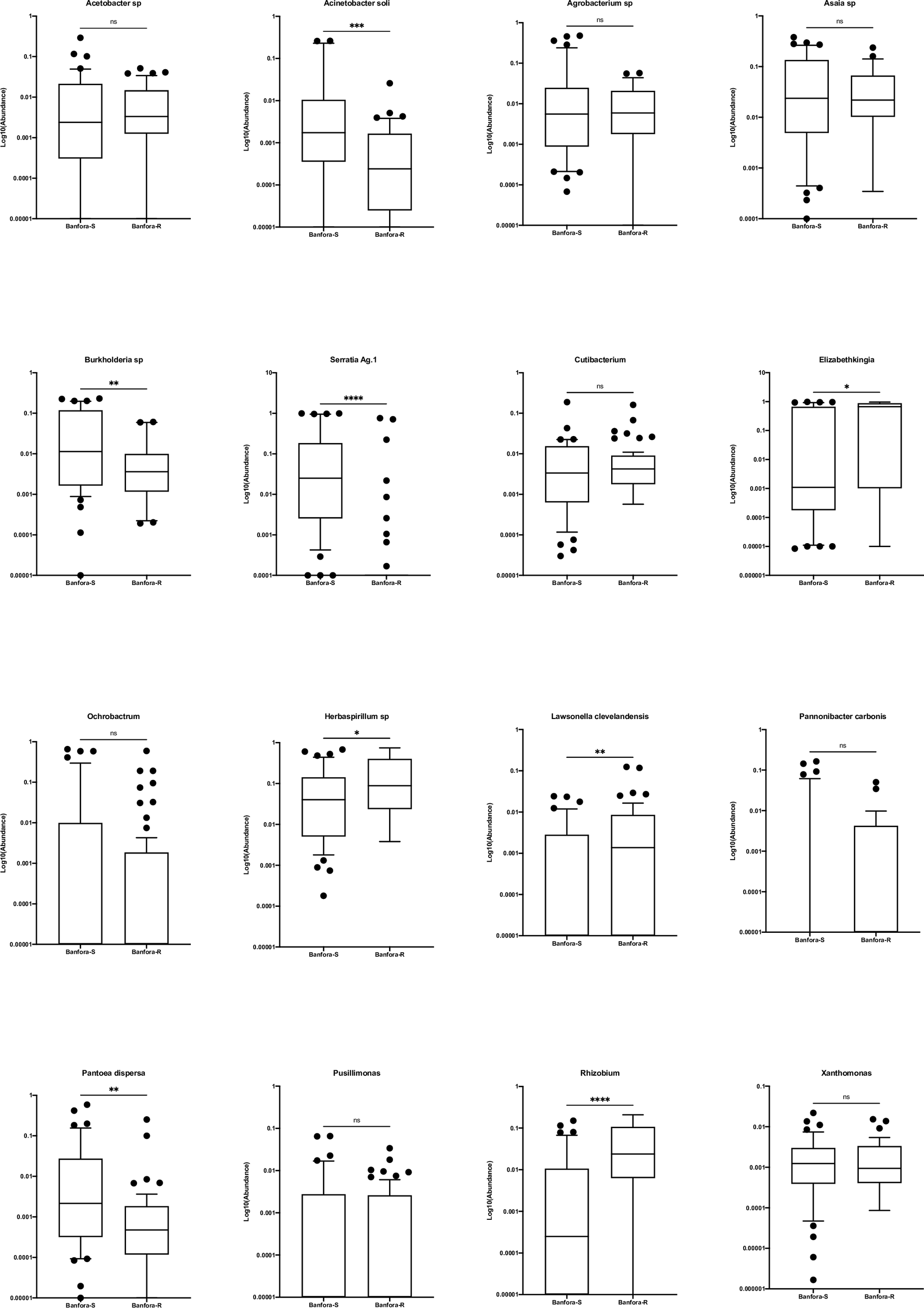

Supplement: S5 Fig — Log10 abundance of each bacteria meeting the cut-off criteria, were compared using a Mann Whitney test. In each case * p < 0.05, ** p < 0.01, *** p < 0.001, **** p < 0.0001. (TIF) [file pgen.1009970.s005.tif]

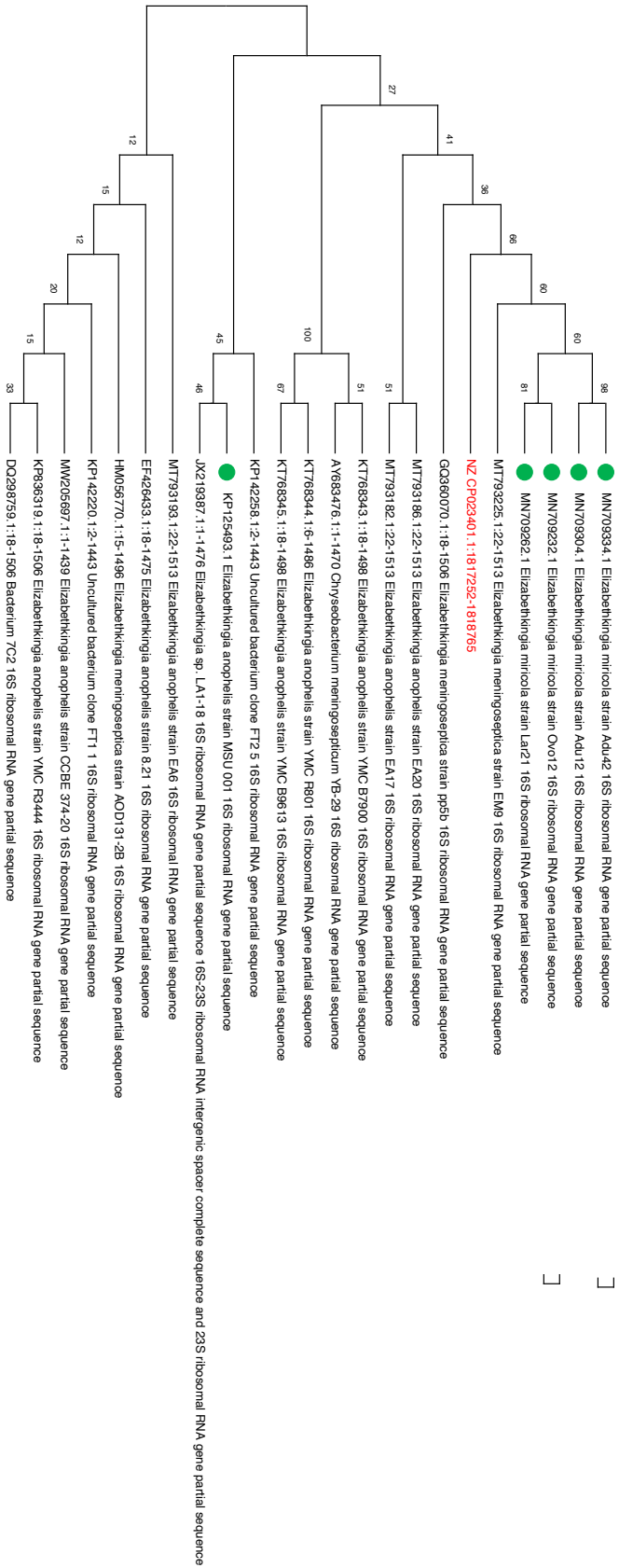

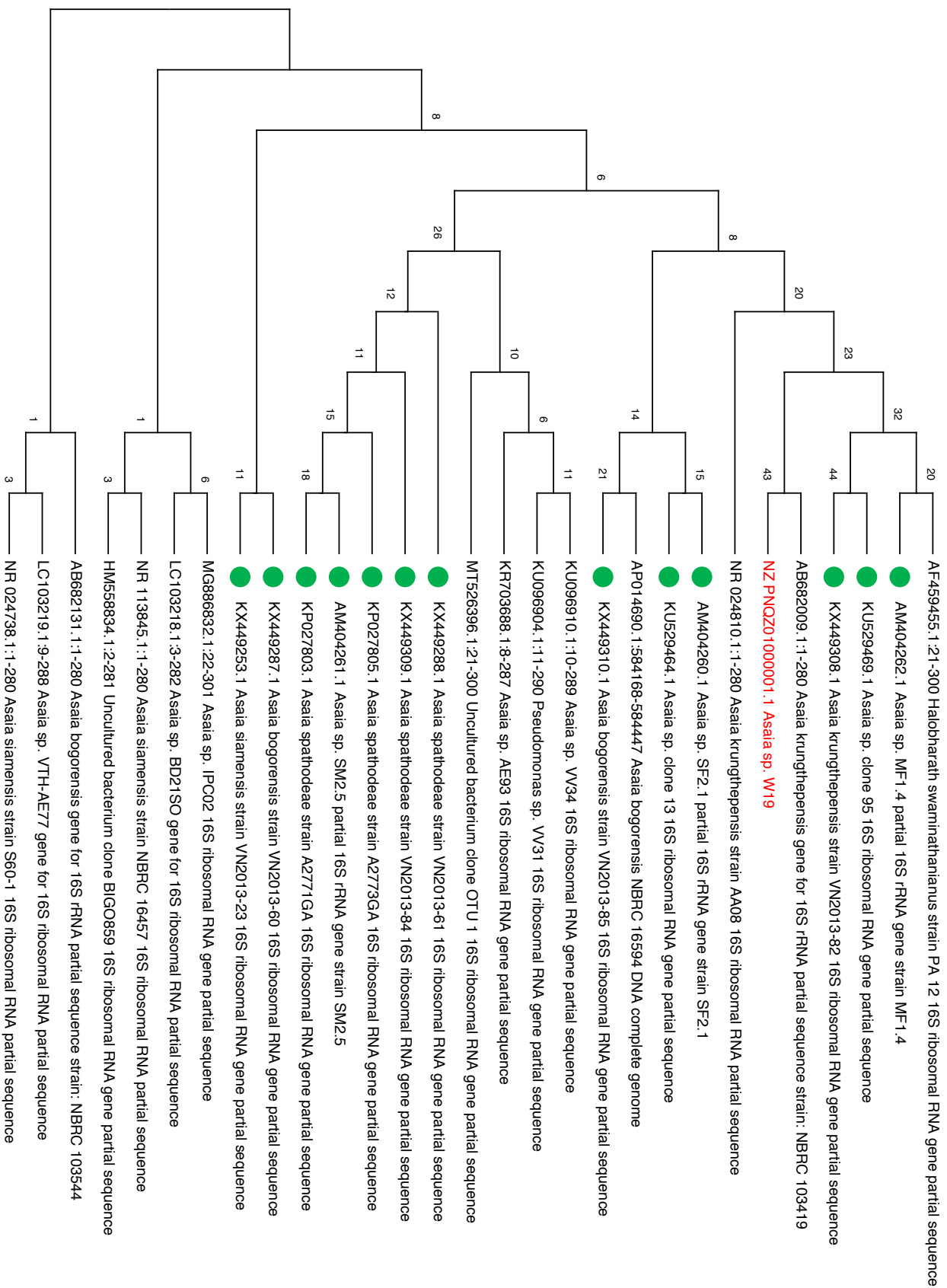

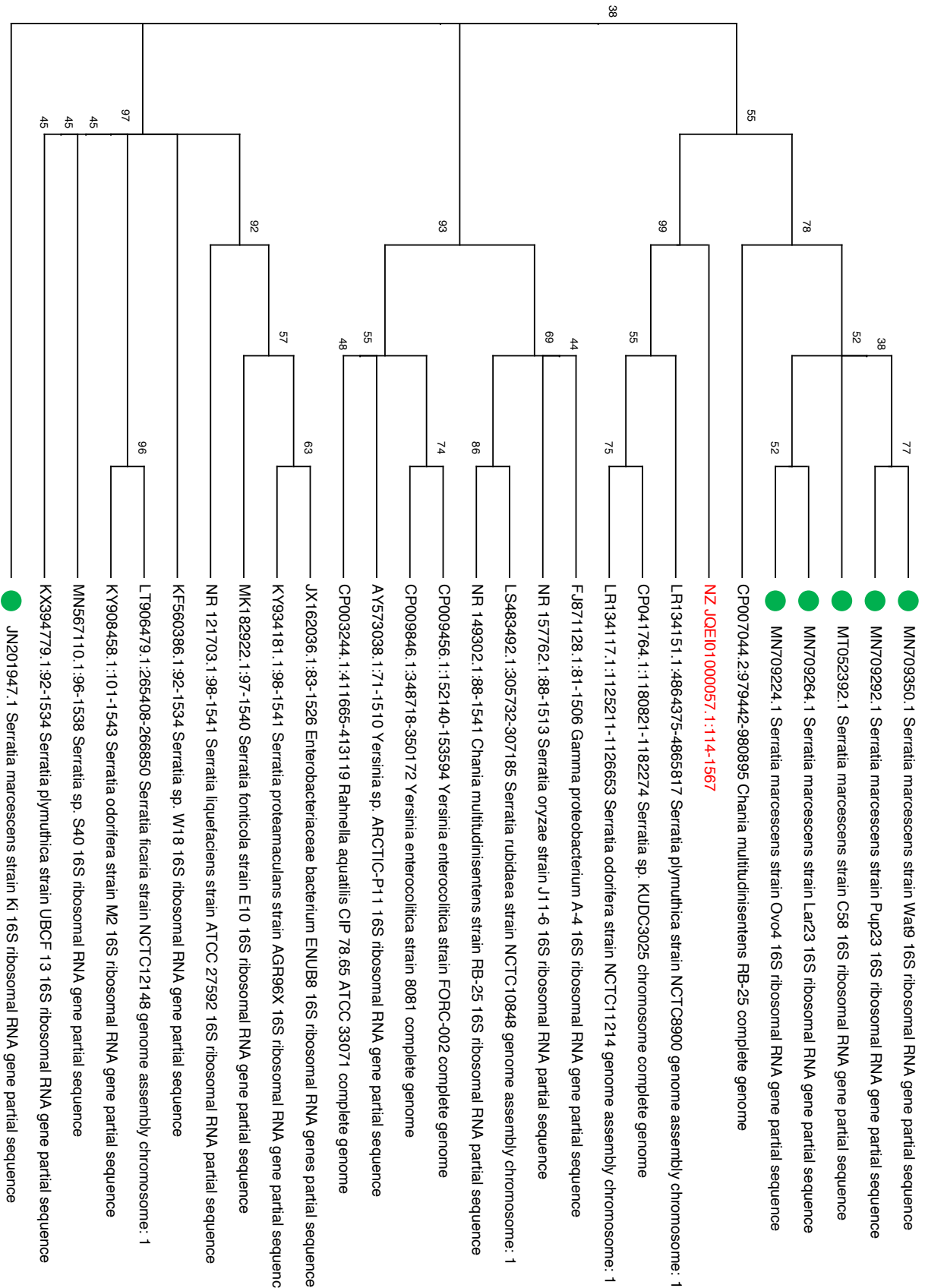

Supplement: S6 Fig — Phylogenies constructed using 16S from the consensus genome for (a) Elizabethkingia; (b) Asaia and (c) Serratia. Each sequence with a green dot is confirmed to have been isolated from an Anopheline mosquito. Sequence extracted from the bacteria in this study are highlighted in red. Sequence names are taken directly from NCBI. Phylogenies created with MegaX using CLUSTAL alignment followed by a Neighbour Joining Tree with 1000 bootstraps. Figures on branches represent bootstrap values. (PDF) [file pgen.1009970.s006.pdf]
