# Supplementary material for: Integration of whole genome sequencing and transcriptomics reveals a complex picture of the reestablishment of insecticide resistance in the major malaria vector Anopheles coluzzii
Source: PLoS Genet. 2021 Dec 23;17(12):e1009970. doi: 10.1371/journal.pgen.1009970 (PMC8741062; doi:10.1371/journal.pgen.1009970)
Supplement: Attachment — Submitted filename: Response to reviewers.docx [file pgen.1009970.s015.docx]

*We thank the reviewers for their positive reviews and suggestions which subsequently improved the manuscript. Below in italics are the responses to each of their questions and comments. In summary, we have expanded the discussion and put more emphasis on the importance/significance of the results.*

Comments to the Authors:
Please note here if the review is uploaded as an attachment.

**Reviewer #1:** The manuscript provides an interesting and in-depth examination of a range of factors that are usually considered separately when examining insecticide resistance. The authors took a sensible research route and had a unique opportunity to examine resistance dynamics which may not be available for examination many times again.

The background provides enough clear detail and context to the study. The methodology is sound, with enough detail to allow replication. The statistical analysis is also described suitably. The mining of whole genome sequencing data is useful, particularly for the microbiome data as this circumvents the shortcomings of 16s sequencing.

My critiques are therefore minor.

1. It is interesting that your resistant strain is physically smaller than the susceptible. This is rather unusual, as it is usually opposite way around and it is thought that vigour tolerance could play a part in resistance phenotype. This should be mentioned at least briefly in the discussion.

*A paragraph has been added that addresses the potential fitness costs resulting in small body size: ‘Within this population, changes in respiratory rate may result in the smaller body size through depleted energetic stores. Although previous studies indicate that larger body size increases vigour and hence resistance, several studies have shown that insecticide resistance and exposure leads to smaller body sizes, mirroring the results of this study.’ Lines 425-429.*

2. Although I think I had understood what you were saying in the text before it, taken on its own, what you are trying to convey in figure 7. The legend does not stand alone and needs to be stated a bit more clearly.

*The figure legend has been rephrased as ‘Figure 7: Selection regime for the susceptible colony. WHO deltamethrin tube selection regime implemented to reselect Banfora S to full resistance. The generations are shown separated by a dashed line. The durations of each exposure to 0.05% deltamethrin are given above the tube, with the mean mortality for each generation below. Mortality for the first two selections are calculated based on entire colony selection (N = 409; N = 514), whilst the final calculation of mortality represents a subset of the selected cage (N = 115).’*

3. Supplementary figure 4: this would benefit from the statistical indicators being present on the Bray Curtis dissimilarity plot.

*As we are unsure which statistical indicators the reviewer is referring to, we have added an approximation of the variance explained by each MDS using the eigenvalue output from the cmdscale function in R.*

4. Although the microbiome data is not the sole focus of the study, I think that it does add to the field of the mosquito microbiome data. I would suggest that one additional supplement would be the inclusion of the associated metadata as listed by the mosquito microbiome project:
<https://mosquito-microbiome.org/files/Metadata_checklist.pdf>

*This extra supplement has been added as Supplementary Table 6 and is mentioned in the methods on line 743 ‘Metadata for the microbiome is detailed in Supplementary Table 6.’.*

5. The one section that I think can be improved is the discussion. While there is nothing wrong with the statements or conclusions, it is a little descriptive, and I think you are missing an opportunity to reflect on a truly wonderful piece of research. What I would have been interested in as a reader (and I would have thought this would have been touched on having just read the abstract) is the significance. I am sure I am not the only mosquito researchers have suspected some of the findings that you have so elegantly displayed. You have shown that transcription changes did not have as dramatic changes in the resistance phenotype as would have been thought. What is the significance of this? Talk about genomic surveillance is everywhere right now. What does your findings mean for genomic surveillance of resistance? Does it have any bearing or does it only apply to your set up. This is just one example, but there is a lot more opportunity for you to get into the meat of your findings, because you have a lot of results to discuss.

*We have expanded the discussion to address the more translational points raised by the reviewer – the changes are highlighted in yellow. These changes address all points the reviewer raises.*

**Reviewer #2:** In this manuscript, the authors used RNAseq and whole genome sequencing to identify changes within the mosquito’s genome, transcriptome and microbiome due to different resistance phenotypes. In the study, a susceptible and two resistant populations, having the same genetic background were used, which, as author claim allow better comparing such variables.

This article is well-written showing few spelling mistakes that can easily be corrected.

In the methods chapter, authors must clarify, at the RNA analysis section, which/how many replicates were in fact used, for each sample. Despite being noticiable in other points of the article it’s necessary to clarify it also here, in the “methods”. Again, at the methods chapter, please identify completely all the reagents and kits used and the same for relevant equipment. The methods and software must be properly referenced.

*The number of individuals used, and number of replicates are now stated in the methods on lines 581-582: ‘5 individuals were used for each of the 4 biological replicates for the RNAseq.’ Kit manufacturer has been included where needed. Software referencing is now complete.*

Regarding the results’s chapter please indicate what does it means “PCA” at the first time it is mentioned.

*PCA has been defined on line 154.*

In order to study the mechanisms of resistance in An. coluzzi, 2 insecticide-resistant populations were used; although, throughout the text there are some references, it is necessary to emphasize the importance of using these two populations. In other words, it must be demonstrated that the inclusion of these populations in the trials was, without a doubt, important and not, on the contrary, unnecessary. So, please mention the relevant differences you found in these two populations showing, as claimed, that their inclusion “allowed a unique comparison of resistant and susceptible mosquitoes with the same genetic background”.

*A sentence has been added in the discussion to emphasise that the Banfora R and Banfora O populations, whilst both being resistant to insecticides, show distinct genomic and transcriptomic profiles (shown in supplementary figure 1 and figure 3), making the use of these two populations critical to ensure processes involved in resistance only are highlighted. Lines 405-407.*

This article reached an excellent amount of results which would expect a more in-depth discussion. Please include more previous works in order to enrich this chapter.

*We have expanded the discussion as requested by both reviewers, this has included more referencing of other relevant works. All changes to the discussion are highlighted in yellow.*

As this paper shows a lot work regarding the changes in resistance restoration, it would benefit the article if this subject was mentioned also in the title.

*The title has been changed to ‘Integration of whole genome sequencing and transcriptomics reveals a complex picture of the reestablishment of insecticide resistance in the major malaria vector Anopheles coluzzii.’*
